# ConSORT: Context- and Flow-Sensitive Ownership Refinement Types for Imperative Programs

**DOI:** 10.1007/978-3-030-44914-8_25

**Published:** 2020-04-18

**Authors:** John Toman, Ren Siqi, Kohei Suenaga, Atsushi Igarashi, Naoki Kobayashi

**Affiliations:** ETH Zurich, Zurich, Switzerland; 9 Kyoto University, Kyoto, Japan; 10 The University of Tokyo, Tokyo, Japan

**Keywords:** Refinement types, Mutable references, Aliasing, Strong updates, Fractional ownerships, Program verification, Type systems

## Abstract

We present ConSORT, a type system for safety verification in the presence of mutability and aliasing. Mutability requires *strong updates* to model changing invariants during program execution, but aliasing between pointers makes it difficult to determine which invariants must be updated in response to mutation. Our type system addresses this difficulty with a novel combination of refinement types and fractional ownership types. Fractional ownership types provide flow-sensitive and precise aliasing information for reference variables. ConSORT interprets this ownership information to soundly handle strong updates of potentially aliased references. We have proved ConSORT sound and implemented a prototype, fully automated inference tool. We evaluated our tool and found it verifies non-trivial programs including data structure implementations.

### 1 Introduction

Driven by the increasing power of automated theorem provers and recent high-profile software failures, fully automated program verification has seen a surge of interest in recent years [5, 10, 15, 29, 38, 66]. In particular, *refinement types* [9, 21, 24, 65], which refine base types with logical predicates, have been shown to be a practical approach for program verification that are amenable to (sometimes full) automation [47, 61, 62, 63]. Despite promising advances [26, 32, 46], the sound and precise application of refinement types (and program verification in general) in settings with mutability and aliasing (e.g., Java, Ruby, etc.) remains difficult.

One of the major challenges is how to precisely and soundly support *strong updates* for the invariants on memory cells. In a setting with mutability, a single invariant may not necessarily hold throughout the lifetime of a memory cell; while the program mutates the memory the invariant may change or evolve. To model these changes, a program verifier must support different, incompatible invariants which hold at different points during program execution. Further, precise program verification requires supporting different invariants on distinct pieces of memory.

```
1 mk(n) { mkref n }
2
3 let p = mk(3) in
4 let q = mk(5) in
5 p := *p + 1;
6 q := *q + 1;
7 assert(*p = 4);
```

**Fig. 1.** Example demonstrating the difficulty of effecting strong updates in the presence of aliasing. The function `mk` is bound in the program from lines 3 to 7; its body is given within the braces.

```
1  loop(a, b) {
2    let aold = *a in
3    b := *b + 1;
4    a := *a + 1;
5    assert(*a = aold + 1);
6    if ⋆ then
7      loop(b, mkref ⋆)
8    else
9      loop(b,a)
10 }
11 loop(mkref ⋆, mkref ⋆)
```

**Fig. 2.** Example with non-trivial aliasing behavior.

One solution is to use refinement types on the static program names (i.e., variables) which point to a memory location. This approach can model evolving invariants while tracking distinct invariants for each memory cell. For example, consider the (contrived) example in Figure 1. This program is written in an ML-like language with mutable references; references are updated with `:=` and allocated with **mkref**. Variable `p` can initially be given the type $\{\nu : \textbf{int} \mid \nu = 3\}\,\textbf{ref}$, indicating it is a reference to the integer 3. Similarly, `q` can be given the type $\{\nu : \textbf{int} \mid \nu = 5\}\,\textbf{ref}$. We can model the mutation of `p`'s memory on line 5 by strongly updating `p`'s type to $\{\nu : \textbf{int} \mid \nu = 4\}\,\textbf{ref}$.

Unfortunately, the precise application of this technique is confounded by the existence of unrestricted aliasing. In general, updating just the type of the mutated reference is insufficient: due to aliasing, other variables may point to the mutated memory and their refinements must be updated as well. However, in the presence of conditional, *may* aliasing, it is impossible to strongly update the refinements on all possible aliases; given the static uncertainty about whether a variable points to the mutated memory, that variable's refinement may only be *weakly updated*. For example, suppose we used a simple alias analysis that imprecisely (but soundly) concluded all references allocated at the same program point *might* alias. Variables `p` and `q` share the allocation site on line 1, so on line 5 we would have to weakly update `q`'s type to $\{\nu : \textbf{int} \mid \nu = 4 \vee \nu = 5\}$, indicating it may hold either 4 *or* 5. Under this same imprecise aliasing assumption, we would also have to weakly update `p`'s type on line 6, preventing the verification of the example program.

Given the precision loss associated with weak updates, it is critical that verification techniques built upon refinement types use precise aliasing information and avoid spuriously applied weak updates. Although it is relatively simple to conclude that `p` and `q` do not alias in Figure 1, consider the example in Figure 2. (In this example, $\star$ represents non-deterministic values.) Verifying this program requires proving `a` and `b` never alias at the writes on lines 3 and 4. In fact, `a` and `b` *may* point to the same memory location, but only in different invocations of `loop`; this pattern may confound even sophisticated symbolic alias analyses.

Additionally, `a` and `b` share an allocation site on line 7, so an approach based on the simple alias analysis described above will also fail on this example. This must-not alias proof obligation *can* be discharged with existing techniques [53, 54], but requires an expensive, on-demand, interprocedural, flow-sensitive alias analysis.

This paper presents ConSORT (CONtext Sensitive Ownership Refinement Types), a type system for the automated verification of program safety in imperative languages with mutability and aliasing. ConSORT is built upon the novel combination of refinement types and fractional ownership types [55, 56]. Fractional ownership types extend pointer types with a rational number in the range $[0, 1]$ called an *ownership*. These ownerships encapsulate the permission of the reference; only references with ownership 1 may be used for mutation. Fractional ownership types also obey the following key invariant: any references with a mutable alias must have ownership 0. Thus, any reference with non-zero ownership *cannot* be an alias of a reference with ownership 1. In other words, ownerships encode precise aliasing information in the form of *must-not* aliasing relationships.

To understand the benefit of this approach, let us return to Figure 1. As `mk` returns a freshly allocated reference with no aliases, its type indicates it returns a reference with ownership 1. Thus, our type system can initially give `p` and `q` types $\{\nu : \textbf{int} \mid \nu = 3\}\,\textbf{ref}^1$ and $\{\nu : \textbf{int} \mid \nu = 5\}\,\textbf{ref}^1$ respectively. The ownership 1 on the reference type constructor **ref** indicates both pointers hold "exclusive" ownership of the pointed to reference cell; from the invariant of fractional ownership types `p` and `q` must *not* alias. The types of both references can be strongly updated *without* requiring spurious weak updates. As a result, at the assertion statement on line 7, `p` has type $\{\nu : \textbf{int} \mid \nu = 4\}\,\textbf{ref}^1$ expressing the required invariant.

Our type system can also verify the example in Figure 2 *without* expensive side analyses. As `a` and `b` are both mutated, they must both have ownership 1; i.e., they cannot alias. This pre-condition is satisfied by all invocations of `loop`; on line 7, `b` has ownership 1 (from the argument type), and the newly allocated reference must also have ownership 1. Similarly, both arguments on line 9 have ownership 1 (from the assumed ownership on the argument types).

Ownerships behave linearly; they cannot be duplicated, only *split* when aliases are created. This linear behavior preserves the critical ownership invariant. For example, if we replace line 9 in Figure 2 with `loop(b,b)`, the program becomes ill-typed; there is no way to divide `b`'s ownership of 1 to into *two* ownerships of 1.

Ownerships also obviate updating refinement information of aliases at mutation. ConSORT ensures that only the trivial refinement $\top$ is used in reference types with ownership 0, i.e., mutably-aliased references. When memory is mutated through a reference with ownership 1, ConSORT simply updates the refinement of the mutated reference variable. From the soundness of ownership types, all aliases have ownership 0 and must therefore only contain the $\top$ refinement. Thus, the types of all aliases already soundly describe *all* possible contents.[3]

ConSORT is also *context-sensitive*, and can use different summaries of function behavior at different points in the program. For example, consider the variant

[3] This assumption holds only if updates do not change simple types, a condition our type-system enforces.

```
1 get(p) { *p }

3 let p = mkref 3 in
4 let q = mkref 5 in
5 p := get(p) + 1;
6 q := get(q) + 1;
7 assert(*p = 4);
8 assert(*q = 6);
```

**Fig. 3.** Example of context-sensitivity of Figure 1 shown in Figure 3. The function `get` returns the contents of its argument, and is called on lines 5 and 6. To precisely verify this program, on line 5 `get` must be typed as a function that takes a reference to 3 and returns 3. Similarly, on line 6 `get` must be typed as a function that takes a reference to 5 and returns 5. Our type system can give `get` a function type that distinguishes between these two calling contexts and selects the appropriate summary of `get`'s behavior.

We have formalized ConSORT as a type system for a small imperative calculus and proved the system is sound: i.e., a well-typed program never encounters assertion failures during execution. We have implemented a prototype type inference tool targeting this imperative language and found it can automatically verify several non-trivial programs, including sorted lists and an array list data structure.

The rest of this paper is organized as follows. Section 2 defines the imperative language targeted by ConSORT and its semantics. Section 3 defines our type system and states our soundness theorem. Section 4 sketches our implementation's inference algorithm and its current limitations. Section 5 describes an evaluation of our prototype, Section 6 outlines related work, and Section 7 concludes.

### 2 Target Language

This section describes a simple imperative language with mutable references and first-order, recursive functions.

#### 2.1 Syntax

We assume a set of *variables*, ranged over by $x, y, z, \ldots$, a set of *function names*, ranged over by $f$, and a set of *labels*, ranged over by $\ell_1, \ell_2, \ldots$. The grammar of the language is as follows.

$$
\begin{array}{rl}
d ::= & f \mapsto (x_1, \ldots, x_n) e \\
e ::= & x \mid \mathbf{let}\, x = y \,\mathbf{in}\, e \mid \mathbf{let}\, x = n \,\mathbf{in}\, e \mid \mathbf{ifz}\, x \,\mathbf{then}\, e_1 \,\mathbf{else}\, e_2 \\
\mid & \mathbf{let}\, x = \mathbf{mkref}\, y \,\mathbf{in}\, e \mid \mathbf{let}\, x = *y \,\mathbf{in}\, e \mid \mathbf{let}\, x = f^{\ell}(y_1, \ldots, y_n) \,\mathbf{in}\, e \\
\mid & x := y \,;\, e \mid \mathbf{alias}(x = y) \,;\, e \mid \mathbf{alias}(x = *y) \,;\, e \mid \mathbf{assert}(\varphi) \,;\, e \mid e_1 \,;\, e_2 \\
P ::= & \langle \{d_1, \ldots, d_n\}, e \rangle
\end{array}
$$

$\varphi$ stands for a formula in propositional first-order logic over variables, integers and contexts; we discuss these formulas later in Section 3.1.

Variables are introduced by function parameters or let bindings. Like ML, the variable bindings introduced by let expressions and parameters are immutable. Mutable variable declarations such as `int x = 1;` in C are achieved in our language with:

$$\mathbf{let}\, y = 1 \,\mathbf{in} (\mathbf{let}\, x = \mathbf{mkref}\, y \,\mathbf{in} \ldots) .$$

As a convenience, we assume all variable names introduced with let bindings and function parameters are distinct.

Unlike ML (and like C or Java) we do not allow general expressions on the right hand side of let bindings. The simplest right hand forms are a variable $y$ or an integer literal $n$. **mkref** $y$ creates a reference cell with value $y$, and $*y$ accesses the contents of reference $y$. For simplicity, we do not include an explicit null value; an extension to support null is discussed in Section 4. Function calls must occur on the right hand side of a variable binding and take the form $f^{\ell}(x_1, \ldots, x_n)$, where $x_1, \ldots, x_n$ are distinct variables and $\ell$ is a (unique) label. These labels are used to make our type system context-sensitive as discussed in Section 3.3.

The single base case for expressions is a single variable. If the variable expression is executed in a tail position of a function, then the value of that variable is the return value of the function, otherwise the value is ignored.

The only intraprocedural control-flow operations in our language are if statements. **ifz** checks whether the condition variable $x$ equals zero and chooses the corresponding branch. Loops can be implemented with recursive functions and we do not include them explicitly in our formalism.

Our grammar requires that side-effecting, result-free statements, **assert**$(\varphi)$ **alias**$(x = y)$, **alias**$(x = *y)$ and assignment $x := y$ are followed by a continuation expression. We impose this requirement for technical reasons to ease our formal presentation; this requirement does not reduce expressiveness as dummy continuations can be inserted as needed. The **assert**$(\varphi)\,;e$ form executes $e$ if the predicate $\varphi$ holds in the current state and aborts the program otherwise. **alias**$(x = y)\,;e$ and **alias**$(x = *y)\,;e$ assert a must-aliasing relationship between $x$ and $y$ (resp. $x$ and $*y$) and then execute $e$. **alias** statements are effectively *annotations* that our type system exploits to gain added precision. $x := y\,;e$ updates the contents of the memory cell pointed to by $x$ with the value of $y$. In addition to the above continuations, our language supports general sequencing with $e_1\,;e_2$.

A program is a pair $\langle D, e\rangle$, where $D = \{d_1, \ldots, d_n\}$ is a set of first-order, mutually recursive function definitions, and $e$ is the program entry point. A function definition $d$ maps the function name to a tuple of argument names $x_1, \ldots, x_n$ that are bound within the function body $e$.

*Paper Syntax.* In the remainder of the paper, we will write programs that are technically illegal according to our grammar, but can be easily "de-sugared" into an equivalent, valid program. For example, we will write

```
let x = mkref 4 in assert(*x = 4)
```

as syntactic sugar for:

```
let f = 4 in let x = mkref f in
let tmp = *x in assert(tmp = 4); let dummy = 0 in dummy
```

### 2.2 Operational Semantics

We now introduce the operational semantics for our language. We assume a finite domain of heap addresses **Addr**: we denote an arbitrary address with $a$.

$$\frac{}{\left\langle H, R, F : \vec{F}, x \right\rangle \longrightarrow_D \left\langle H, R, \vec{F}, F[x] \right\rangle} \text{ (R-Var)} \qquad \frac{}{\left\langle H, R, F : \vec{F}, E[x\,;e] \right\rangle \longrightarrow_D \left\langle H, R, \vec{F}, E[e] \right\rangle} \text{ (R-Seq)}$$

$$\frac{x' \notin dom(R)}{\left\langle H, R, \vec{F}, E[\mathbf{let}\, x = y\, \mathbf{in}\, e] \right\rangle \longrightarrow_D \left\langle H, R\{x' \mapsto R(y)\}, \vec{F}, E[[x'/x]e] \right\rangle} \text{ (R-Let)}$$

$$\frac{x' \notin dom(R)}{\left\langle H, R, \vec{F}, E[\mathbf{let}\, x = n\, \mathbf{in}\, e] \right\rangle \longrightarrow_D \left\langle H, R\{x' \mapsto n\}, \vec{F}, E[[x'/x]e] \right\rangle} \text{ (R-LetInt)}$$

$$\frac{R(x) = 0}{\left\langle H, R, \vec{F}, E[\mathbf{ifz}\, x\, \mathbf{then}\, e_1\, \mathbf{else}\, e_2] \right\rangle \longrightarrow_D \left\langle H, R, \vec{F}, E[e_1] \right\rangle} \text{ (R-IfTrue)}$$

$$\frac{R(x) \neq 0}{\left\langle H, R, \vec{F}, E[\mathbf{ifz}\, x\, \mathbf{then}\, e_1\, \mathbf{else}\, e_2] \right\rangle \longrightarrow_D \left\langle H, R, \vec{F}, E[e_2] \right\rangle} \text{ (R-IfFalse)}$$

$$\frac{a \notin dom(H) \qquad x' \notin dom(R)}{\left\langle H, R, \vec{F}, E[\mathbf{let}\, x = \mathbf{mkref}\, y\, \mathbf{in}\, e] \right\rangle \longrightarrow_D \left\langle H\{a \mapsto R(y)\}, R\{x' \mapsto a\}, \vec{F}, E[[x'/x]e] \right\rangle} \text{ (R-MkRef)}$$

$$\frac{R(y) = a \qquad H(a) = v \qquad x' \notin dom(R)}{\left\langle H, R, \vec{F}, E[\mathbf{let}\, x = *y\, \mathbf{in}\, e] \right\rangle \longrightarrow_D \left\langle H, R\{x' \mapsto v\}, \vec{F}, E[[x'/x]e] \right\rangle} \text{ (R-Deref)}$$

**Fig. 4.** Transition Rules (1).

A runtime state is represented by a configuration $\left\langle H, R, \vec{F}, e \right\rangle$, which consists of a heap, register file, stack, and currently reducing expression respectively. The register file maps variables to runtime values $v$, which are either integers $n$ or addresses $a$. The heap maps a finite subset of addresses to runtime values. The runtime stack represents pending function calls as a sequence of return contexts, which we describe below. While the final configuration component is an expression, the rewriting rules are defined in terms of $E[e]$, which is an evaluation context $E$ and redex $e$, as is standard. The grammar for evaluation contexts is defined by: $E ::= E'\,; e \mid [\,]$.

Our operational semantics is given in Figures 4 and 5. We write $dom(H)$ to indicate the domain of a function and $H\{a \mapsto v\}$ where $a \notin dom(H)$ to denote a map which takes all values in $dom(H)$ to their values in $H$ and which additionally takes $a$ to $v$. We will write $H\{a \hookleftarrow v\}$ where $a \in dom(H)$ to denote a map equivalent to $H$ except that $a$ takes value $v$. We use similar notation for $dom(R)$ and $R\{x \mapsto v\}$. We also write $\emptyset$ for the empty register file and heap. The step relation $\longrightarrow_D$ is parameterized by a set of function definitions $D$; a program $\langle D, e\rangle$ is executed by stepping the initial configuration $\langle \emptyset, \emptyset, \cdot, e\rangle$ according to $\longrightarrow_D$. The semantics is mostly standard; we highlight some important points below.

Return contexts $F$ take the form $E[\mathbf{let}\, y = [\,]^\ell\, \mathbf{in}\, e]$. A return context represents a pending function call with label $\ell$, and indicates that $y$ should be bound to the return value of the callee during the execution of $e$ within the larger execution context $E$. The call stack $\vec{F}$ is a sequence of these contexts, with the first such return context representing the most recent function call. The stack grows at function calls as described by rule R-Call. For a call $E[\mathbf{let}\, x = f^\ell(y_1, \ldots, y_n)\, \mathbf{in}\, e]$ where $f$ is defined as $(x_1, \ldots, x_n)e'$, the return context $E[\mathbf{let}\, y = [\,]^\ell\, \mathbf{in}\, e]$ is

$$\frac{f \mapsto (x_1, .., x_n)e \in D}{\left\langle H, R, \vec{F}, E[\mathbf{let}\, x = f^{\ell}(y_1, \ldots, y_n)\, \mathbf{in}\, e'] \right\rangle \longrightarrow_D \left\langle H, R, E[\mathbf{let}\, x = []^{\ell}\, \mathbf{in}\, e'] : \vec{F}, [y_1/x_1] \cdots [y_n/x_n] e \right\rangle} \text{(R-Call)}$$

$$\frac{R(x) = a \qquad a \in dom(H)}{\left\langle H, R, \vec{F}, E[x := y\, ;\, e] \right\rangle \longrightarrow_D \left\langle H\{a \hookleftarrow R(y)\}, R, \vec{F}, E[e] \right\rangle} \text{(R-Assign)}$$

$$\frac{R(x) = R(y)}{\left\langle H, R, \vec{F}, E[\mathbf{alias}(x = y)\, ;\, e] \right\rangle \longrightarrow_D \left\langle H, R, \vec{F}, E[e] \right\rangle} \text{(R-Alias)}$$

$$\frac{R(y) = a \qquad H(a) = R(x)}{\left\langle H, R, \vec{F}, E[\mathbf{alias}(x = *y)\, ;\, e] \right\rangle \longrightarrow_D \left\langle H, R, \vec{F}, E[e] \right\rangle} \text{(R-AliasPtr)}$$

$$\frac{R(x) \neq R(y)}{\left\langle H, R, \vec{F}, E[\mathbf{alias}(x = y)\, ;\, e] \right\rangle \longrightarrow_D \mathbf{AliasFail}} \text{(R-AliasFail)}$$

$$\frac{R(x) \neq H(R(y))}{\left\langle H, R, \vec{F}, E[\mathbf{alias}(x = *y)\, ;\, e] \right\rangle \longrightarrow_D \mathbf{AliasFail}} \text{(R-AliasPtrFail)}$$

$$\frac{\models [R]\, \varphi}{\left\langle H, R, \vec{F}, E[\mathbf{assert}(\varphi)\, ;\, e] \right\rangle \longrightarrow_D \left\langle H, R, \vec{F}, E[e] \right\rangle} \text{(R-Assert)}$$

$$\frac{\not\models [R]\, \varphi}{\left\langle H, R, \vec{F}, E[\mathbf{assert}(\varphi)\, ;\, e] \right\rangle \longrightarrow_D \mathbf{AssertFail}} \text{(R-AssertFail)}$$

**Fig. 5.** Transition Rules (2).

prepended onto the stack of the input configuration. The substitution of formal arguments for parameters in $e'$, denoted by $[y_1/x_1] \cdots [y_n/x_n]e'$, becomes the currently reducing expression in the output configuration. Function returns are handled by R-Var. Our semantics return values by name; when the currently executing function fully reduces to a single variable $x$, $x$ is substituted into the return context on the top of the stack, denoted by $E[\mathbf{let}\, y = []^{\ell}\, \mathbf{in}\, e][x]$.

In the rules R-Assert we write $\models [R]\, \varphi$ to mean that the formula yielded by substituting the concrete values in $R$ for the variables in $\varphi$ is valid within some chosen logic (see Section 3.1); in R-AssertFail we write $\not\models [R]\, \varphi$ when the formula is *not* valid. The substitution operation $[R]\, \varphi$ is defined inductively as $[\emptyset]\, \varphi = \varphi$, $[R\{x \mapsto n\}]\, \varphi = [R]\, [n/x]\varphi$, $[R\{x \mapsto a\}]\, \varphi = [R]\, \varphi$. In the case of an assertion failure, the semantics steps to a distinguished configuration **AssertFail**. The goal of our type system is to show that no execution of a well-typed program may reach this configuration. The **alias** form checks whether the two references actually alias; i.e., if the must-alias assertion provided by the programmer is correct. If not, our semantics steps to the distinguished **AliasFail** configuration. Our type system does *not* guarantee that **AliasFail** is unreachable; aliasing assertions are effectively trusted annotations that are assumed to hold.

In order to avoid duplicate variable names in our register file due to recursive functions, we refresh the bound variable $x$ in a let expression to $x'$. Take expression $\mathbf{let}\, x = y\, \mathbf{in}\, e$ as an example; we substitute a fresh variable $x'$ for $x$ in $e$, then bind $x'$ to the value of variable $y$. We assume this refreshing of variables preserves our assumption that all variable bindings introduced with let and function parameters are unique, i.e. $x'$ does not overlap with variable names that occur in the program.

$$
\begin{array}{llcl}
\text{Types} & \tau & ::= & \{\nu : \mathbf{int} \mid \varphi\} \mid \tau\, \mathbf{ref}^r \\
\text{Ownership} & r & \in & [0,1] \\
\text{Refinements} & \varphi & ::= & \varphi_1 \vee \varphi_2 \mid \neg\varphi \mid \top \\
 & & \mid & \phi(\widehat{v}_1, .., \widehat{v}_n) \\
 & & \mid & \widehat{v}_1 = \widehat{v}_2 \\
 & & \mid & \mathcal{CP} \\
\text{Ref. Values} & \widehat{v} & ::= & x \mid n \mid \nu
\end{array}
\qquad
\begin{array}{llcl}
\text{Function Types} & \sigma & ::= & \forall\lambda.\, \langle x_1 : \tau_1, \ldots, x_n : \tau_n \rangle \\
 & & & \to \langle x_1 : \tau_1', \ldots, x_n : \tau_n' \mid \tau \rangle \\
\text{Context Variables} & \lambda & \in & \mathbf{CVar} \\
\text{Concrete Context} & \vec{\ell} & ::= & \ell : \vec{\ell} \mid \epsilon \\
\text{Pred. Context} & \mathcal{C} & ::= & \ell : \mathcal{C} \mid \lambda \mid \epsilon \\
\text{Context Query} & \mathcal{CP} & ::= & \vec{\ell} \preceq \mathcal{C} \\
\text{Typing Context} & \mathcal{L} & ::= & \lambda \mid \vec{\ell}
\end{array}
$$

**Fig. 6.** Syntax of types, refinements, and contexts.

## 3 Typing

We now introduce a fractional ownership refinement type system that guarantees well-typed programs do not encounter assertion failures.

### 3.1 Types and Contexts

The syntax of types is given in Figure 6. Our type system has two type constructors: references and integers. $\tau\,\mathbf{ref}^r$ is the type of a (non-null) reference to a value of type $\tau$. $r$ is an ownership which is a rational number in the range $[0, 1]$. An ownership of 0 indicates a reference that cannot be written, and for which there may exist a mutable alias. By contrast, 1 indicates a pointer with exclusive ownership that can be read and written. Reference types with ownership values between these two extremes indicate a pointer that is readable but not writable, and for which no mutable aliases exist. ConSORT ensures that these invariants hold while aliases are created and destroyed during execution.

Integers are refined with a predicate $\varphi$. The language of predicates is built using the standard logical connectives of first-order logic, with (in)equality between variables and integers, and atomic predicate symbols $\phi$ as the basic atoms. We include a special "value" variable $\nu$ representing the value being refined by the predicate. For simplicity, we omit the connectives $\varphi_1 \wedge \varphi_2$ and $\varphi_1 \implies \varphi_2$; they can be written as derived forms using the given connectives. We do not fix a particular theory from which $\phi$ are drawn, provided a sound (but not necessarily complete) decision procedure exists. $\mathcal{CP}$ are context predicates, which are used for context sensitivity as explained below.

*Example 1.* $\{\nu : \mathbf{int} \mid \nu > 0\}$ is the type of strictly positive integers. The type of immutable references to integers exactly equal to 3 can be expressed by $\{\nu : \mathbf{int} \mid \nu = 3\}\,\mathbf{ref}^{0.5}$.

As is standard, we denote a type environment with $\Gamma$, which is a finite map from variable names to type $\tau$. We write $\Gamma[x : \tau]$ to denote a type environment $\Gamma$ such that $\Gamma(x) = \tau$ where $x \in dom(\Gamma)$, $\Gamma, x : \tau$ to indicate the extension of $\Gamma$ with the type binding $x : \tau$, and $\Gamma[x \hookleftarrow \tau]$ to indicate the type environment $\Gamma$ with the binding of $x$ updated to $\tau$. We write the empty environment as

•. The treatment of type environments as mappings instead of sequences in a dependent type system is somewhat non-standard. The standard formulation based on ordered sequences of bindings and its corresponding well-formedness condition did not easily admit variables with mutually dependent refinements as introduced by our function types (see below). We therefore use an unordered environment and relax well-formedness to ignore variable binding order.

*Function Types, Contexts, and Context Polymorphism.* Our type system achieves context sensitivity by allowing function types to depend on where a function is called, i.e., the *execution context* of the function invocation. Our system represents a *concrete* execution contexts with strings of call site labels (or just "call strings"), defined by $\vec{\ell} ::= \epsilon \mid \ell : \vec{\ell}$. As is standard (e.g., [49, 50]), the string $\ell : \vec{\ell}$ abstracts an execution context where the most recent, active function call occurred at call site $\ell$ which itself was executed in a context abstracted by $\vec{\ell}$; $\epsilon$ is the context under which program execution begins. *Context variables*, drawn from a finite domain **CVar** and ranged over by $\lambda_1, \lambda_2, \ldots$, represent arbitrary, unknown contexts.

A function type takes the form $\forall \lambda. \langle x_1 : \tau_1, \ldots, x_n : \tau_n \rangle \rightarrow \langle x_1 : \tau'_1, \ldots, x_n : \tau'_n \mid \tau \rangle$. The arguments of a function are an $n$-ary tuple of types $\tau_i$. To model side-effects on arguments, the function type includes the same number of *output types* $\tau'_i$. In addition, function types have a direct return type $\tau$. The argument and output types are given names: refinements within the function type may refer to these names. Function types in our language are context polymorphic, expressed by universal quantification "$\forall\lambda.$" over a context variable. Intuitively, this context variable represents the many different execution contexts under which a function may be called.

Argument and return types may depend on this context variable by including *context query predicates* in their refinements. A context query predicate $\mathcal{CP}$ usually takes the form $\vec{\ell} \preceq \lambda$, and is true iff $\vec{\ell}$ is a prefix of the concrete context represented by $\lambda$. Intuitively, a refinement $\vec{\ell} \preceq \lambda \implies \varphi$ states that $\varphi$ holds in any concrete execution context with prefix $\vec{\ell}$, and provides no information in any other context. In full generality, a context query predicate may be of the form $\vec{\ell_1} \preceq \vec{\ell_2}$ or $\vec{\ell} \preceq \ell_1 \ldots \ell_n : \lambda$; these forms may be immediately simplified to $\top$, $\bot$ or $\vec{\ell'} \preceq \lambda$.

*Example 2.* The type $\{\nu : \mathbf{int} \mid (\ell_1 \preceq \lambda \implies \nu = 3) \wedge (\ell_2 \preceq \lambda \implies \nu = 5)\}$ represents an integer that is 3 if the most recent active function call site is $\ell_1$, 5 if the most recent call site is $\ell_2$, and is otherwise unconstrained. This type may be used for the argument of `f` in, e.g., $\mathtt{f}^{\ell_1}\mathtt{(3)}\ \mathtt{+}\ \mathtt{f}^{\ell_2}\mathtt{(5)}$.

As types in our type system may contain context variables, our typing judgment (introduced below) includes a typing context $\mathcal{L}$, which is either a single context variable $\lambda$ or a concrete context $\vec{\ell}$. This typing context represents the assumptions about the execution context of the term being typed. If the typing context is a context variable $\lambda$, then no assumptions are made about the execution context of the term, although types may depend upon $\lambda$ with context query predicates. Accordingly, function bodies are typed under the context variable universally quantified over in the corresponding function type; i.e., no assumptions are made about the exact execution context of the function body.

As in parametric polymorphism, consistent substitution of a concrete context $\vec{\ell}$ for a context variable $\lambda$ in a typing derivation yields a valid type derivation under concrete context $\vec{\ell}$.

*Remark 1.* The context-sensitivity scheme described here corresponds to the standard CFA approach [50] without *a priori* call-string limiting. We chose this scheme because it can be easily encoded with equality over integer variables (see Section 4), but in principle another context-sensitivity strategy could be used instead. The important feature of our type system is the inclusion of predicates over contexts, not the specific choice for these predicates.

Function type environments are denoted with $\Theta$ and are finite maps from function names ($f$) to function types ($\sigma$).

*Well Formedness.* We impose two well-formedness conditions on types: *ownership well-formedness* and *refinement well-formedness*. The ownership condition is purely syntactic: $\tau$ is ownership well-formed if $\tau = \tau' \, \mathbf{ref}^0$ implies $\tau' = \top_n$ for some $n$. $\top_i$ is the "maximal" type of a chain of $i$ references, and is defined inductively as $\top_0 = \{\nu : \mathbf{int} \mid \top\}$, $\top_i = \top_{i-1} \, \mathbf{ref}^0$.

The ownership well-formedness condition ensures that aliases introduced via heap writes do not violate the invariant of ownership types *and* that refinements are consistent with updates performed through mutable aliases. Recall our ownership type invariant ensures all aliases of a mutable reference have 0 ownership. Any mutations through that mutable alias will therefore be consistent with the "no information" $\top$ refinement required by this well-formedness condition.

Refinement well-formedness, denoted $\mathcal{L} \mid \Gamma \vdash_{WF} \varphi$, ensures that free program variables in refinement $\varphi$ are bound in a type environment $\Gamma$ and have integer type. It also requires that for a typing context $\mathcal{L} = \lambda$, only context query predicates over $\lambda$ are used (no such predicates may be used if $\mathcal{L} = \vec{\ell}$). Notice this condition forbids refinements that refer to references. Although ownership information can signal when refinements on a mutably-aliased reference must be discarded, our current formulation provides no such information for refinements that *mention* mutably-aliased references. We therefore conservatively reject such refinements at the cost of some expressiveness in our type system.

We write $\mathcal{L} \mid \Gamma \vdash_{WF} \tau$ to indicate a well-formed type where all refinements are well-formed with respect to $\mathcal{L}$ and $\Gamma$. We write $\mathcal{L} \vdash_{WF} \Gamma$ for a type environment where all types are well-formed. A function environment is well-formed (written $\vdash_{WF} \Theta$) if, for every $\sigma$ in $\Theta$, the argument, result, and output types are well-formed with respect to each other and the context variable quantified over in $\sigma$. As the formal definition of refinement well-formedness is fairly standard, we omit it for space reasons (the full definition may be found in the full version [60]).

#### 3.2 Intraprocedural Type System

We now introduce the type system for the intraprocedural fragment of our language. Accordingly, this section focuses on the interplay of mutability and

$$\frac{}{\Theta \mid \mathcal{L} \mid \Gamma[x : \tau_1 + \tau_2] \vdash x : \tau_1 \Rightarrow \Gamma[x \hookleftarrow \tau_2]} \quad \text{(T-Var)}$$

$$\frac{\Theta \mid \mathcal{L} \mid \Gamma[y \hookleftarrow \tau_1 \wedge_y y =_{\tau_1} x], x : (\tau_2 \wedge_x x =_{\tau_2} y) \vdash e : \tau \Rightarrow \Gamma' \qquad x \notin dom(\Gamma')}{\Theta \mid \mathcal{L} \mid \Gamma[y : \tau_1 + \tau_2] \vdash \mathbf{let}\, x = y\, \mathbf{in}\, e : \tau \Rightarrow \Gamma'} \quad \text{(T-Let)}$$

$$\frac{\Theta \mid \mathcal{L} \mid \Gamma, x : \{\nu : \mathbf{int} \mid \nu = n\} \vdash e : \tau \Rightarrow \Gamma' \qquad x \notin dom(\Gamma')}{\Theta \mid \mathcal{L} \mid \Gamma \vdash \mathbf{let}\, x = n\, \mathbf{in}\, e : \tau \Rightarrow \Gamma'} \quad \text{(T-LetInt)}$$

$$\frac{\begin{array}{c}\Theta \mid \mathcal{L} \mid \Gamma[x \hookleftarrow \{\nu : \mathbf{int} \mid \varphi \wedge \nu = 0\}] \vdash e_1 : \tau \Rightarrow \Gamma' \\ \Theta \mid \mathcal{L} \mid \Gamma[x \hookleftarrow \{\nu : \mathbf{int} \mid \varphi \wedge \nu \neq 0\}] \vdash e_2 : \tau \Rightarrow \Gamma'\end{array}}{\Theta \mid \mathcal{L} \mid \Gamma[x : \{\nu : \mathbf{int} \mid \varphi\}] \vdash \mathbf{ifz}\, x\, \mathbf{then}\, e_1\, \mathbf{else}\, e_2 : \tau \Rightarrow \Gamma'} \quad \text{(T-If)}$$

$$\frac{\begin{array}{c}\Theta \mid \mathcal{L} \mid \Gamma[y \hookleftarrow \tau_1], x : (\tau_2 \wedge_x x =_{\tau_2} y)\, \mathbf{ref}^1 \vdash e : \tau \Rightarrow \Gamma' \\ x \notin dom(\Gamma')\end{array}}{\Theta \mid \mathcal{L} \mid \Gamma[y : \tau_1 + \tau_2] \vdash \mathbf{let}\, x = \mathbf{mkref}\, y\, \mathbf{in}\, e : \tau \Rightarrow \Gamma'} \quad \text{(T-MkRef)}$$

$$\frac{\begin{array}{c}\Theta \mid \mathcal{L} \mid \Gamma \vdash e_1 : \tau' \Rightarrow \Gamma' \\ \Theta \mid \mathcal{L} \mid \Gamma' \vdash e_2 : \tau'' \Rightarrow \Gamma''\end{array}}{\Theta \mid \mathcal{L} \mid \Gamma \vdash e_1 \,;\, e_2 : \tau'' \Rightarrow \Gamma''} \quad \text{(T-Seq)}$$

$$\frac{\begin{array}{c}\tau' = \begin{cases}\tau_1 \wedge_y y =_{\tau_1} x & r > 0 \\ \tau_1 & r = 0\end{cases} \\ \Theta \mid \mathcal{L} \mid \Gamma[y \hookleftarrow \tau'\, \mathbf{ref}^r], x : \tau_2 \vdash e : \tau \Rightarrow \Gamma' \\ x \notin dom(\Gamma')\end{array}}{\Theta \mid \mathcal{L} \mid \Gamma[y : (\tau_1 + \tau_2)\, \mathbf{ref}^r] \vdash \mathbf{let}\, x = *y\, \mathbf{in}\, e : \tau \Rightarrow \Gamma'} \quad \text{(T-Deref)}$$

$$\frac{\begin{array}{c}\Gamma \models \varphi \qquad \epsilon \mid \Gamma \vdash_{WF} \varphi \\ \Theta \mid \mathcal{L} \mid \Gamma \vdash e : \tau \Rightarrow \Gamma'\end{array}}{\Theta \mid \mathcal{L} \mid \Gamma \vdash \mathbf{assert}(\varphi) \,;\, e : \tau \Rightarrow \Gamma'} \quad \text{(T-Assert)}$$

**Fig. 7.** Expression typing rules.

refinement types. The typing rules are given in Figures 7 and 8. A typing judgment takes the form $\Theta \mid \mathcal{L} \mid \Gamma \vdash e : \tau \Rightarrow \Gamma'$, which indicates that $e$ is well-typed under a function type environment $\Theta$, typing context $\mathcal{L}$, and type environment $\Gamma$, and evaluates to a value of type $\tau$ and modifies the input environment according to $\Gamma'$. Any valid typing derivation must have $\mathcal{L} \vdash_{WF} \Gamma$, $\mathcal{L} \vdash_{WF} \Gamma'$, and $\mathcal{L} \mid \Gamma' \vdash_{WF} \tau$, i.e., the input and output type environments and result type must be well-formed.

The typing rules in Figure 7 handle the relatively standard features in our language. The rule T-Seq for sequential composition is fairly straightforward except that the output type environment for $e_1$ is the input type environment for $e_2$. T-LetInt is also straightforward; since $x$ is bound to a constant, it is given type $\{\nu : \mathbf{int} \mid \nu = n\}$ to indicate $x$ is exactly $n$. The output type environment $\Gamma'$ cannot mention $x$ (expressed with $x \notin dom(\Gamma')$) to prevent $x$ from escaping its scope. This requirement can be met by applying the subtyping rule (see below) to weaken refinements to no longer mention $x$. As in other refinement type systems [47], this requirement is critical for ensuring soundness.

Rule T-Let is crucial to understanding our ownership type system. The body of the let expression $e$ is typechecked under a type environment where the type of $y$ in $\Gamma$ is linearly split into two types: $\tau_1$ for $y$ and $\tau_2$ for the newly created binding $x$. This splitting is expressed using the + operator. If $y$ is a reference type, the split operation distributes some portion of $y$'s ownership information to its new alias $x$. The split operation also distributes refinement information between the two types. For example, type $\{\nu : \mathbf{int} \mid \nu > 0\}\, \mathbf{ref}^1$ can be split into (1) $\{\nu : \mathbf{int} \mid \nu > 0\}\, \mathbf{ref}^r$ and $\{\nu : \mathbf{int} \mid \nu > 0\}\, \mathbf{ref}^{(1-r)}$ (for $r \in (0, 1)$), i.e., two *immutable* references with non-trivial refinement information, or (2) $\{\nu : \mathbf{int} \mid \nu > 0\}\,\mathbf{ref}^1$ and $\{\nu : \mathbf{int} \mid \top\}\,\mathbf{ref}^0$, where one of the aliases is mutable and the other provides no refinement information. How a type is split depends on the usage of $x$ and $y$ in $e$. Formally, we define the type addition operator as the least commutative partial operation that satisfies the following rules:

$$\{\nu : \mathbf{int} \mid \varphi_1\} + \{\nu : \mathbf{int} \mid \varphi_2\} = \{\nu : \mathbf{int} \mid \varphi_1 \wedge \varphi_2\} \qquad \text{(TADD-INT)}$$

$$\tau_1\,\mathbf{ref}^{r_1} + \tau_2\,\mathbf{ref}^{r_2} = (\tau_1 + \tau_2)\,\mathbf{ref}^{r_1+r_2} \qquad \text{(TADD-REF)}$$

Viewed another way, type addition describes how to combine two types for the same value such that the combination soundly incorporates all information from the two original types. Critically, the type addition operation cannot create or destroy ownership and refinement information, only combine or divide it between types. Although not explicit in the rules, by ownership well-formedness, if the entirety of a reference's ownership is transferred to another type during a split, all refinements in the remaining type must be $\top$.

The additional bits $\wedge_y y =_{\tau_1} x$ and $\wedge_x x =_{\tau_2} y$ express equality between $x$ and $y$ as refinements. We use the strengthening operation $\tau \wedge_x \varphi$ and typed equality proposition $x =_\tau y$, defined respectively as:

$$\{\nu : \mathbf{int} \mid \varphi\} \wedge_y \varphi' = \{\nu : \mathbf{int} \mid \varphi \wedge [\nu/y]\,\varphi'\} \qquad (x =_{\{\nu : \mathbf{int} \mid \varphi\}} y) = (x = y)$$

$$\tau\,\mathbf{ref}^r \wedge_y \varphi' = \tau\,\mathbf{ref}^r \qquad (x =_{\tau\,\mathbf{ref}^r} y) = \top$$

We do not track equality between references or between the contents of aliased reference cells as doing so would violate our refinement well-formedness condition. These operations are also used in other rules that can introduce equality.

Rule T-MkRef is very similar to T-Let, except that $x$ is given a reference type of ownership 1 pointing to $\tau_2$, which is obtained by splitting the type of $y$. In T-Deref, the content type of $y$ is split and distributed to $x$. The strengthening is *conditionally* applied depending on the ownership of the dereferenced pointer, that is, if $r = 0$, $\tau'$ has to be a maximal type $\top_i$.

Our type system also tracks path information; in the T-If rule, we update the refinement on the condition variable within the respective branches to indicate whether the variable must be zero. By requiring both branches to produce the same output type environment, we guarantee that these conflicting refinements are rectified within the type derivations of the two branches.

The type rule for assert statements has the precondition $\Gamma \models \varphi$ which is defined to be $\models [\![\Gamma]\!] \implies \varphi$, i.e., the logical formula $[\![\Gamma]\!] \implies \varphi$ is valid in the chosen theory. $[\![\Gamma]\!]$ lifts the refinements on the integer valued variables into a proposition in the logic used for verification. This denotation operation is defined as:

$$[\![\bullet]\!] = \top \qquad [\![\{\nu : \mathbf{int} \mid \varphi\}]\!]_y = [y/\nu]\,\varphi$$

$$[\![\Gamma, x : \tau]\!] = [\![\Gamma]\!] \wedge [\![\tau]\!]_x \qquad [\![\tau'\,\mathbf{ref}^r]\!]_y = \top$$

If the formula $[\![\Gamma]\!] \implies \varphi$ is valid, then in any context and under any valuation of program variables that satisfy the refinements in $[\![\Gamma]\!]$, the predicate $\varphi$ must be true and the assertion must not fail. This intuition forms the foundation of our soundness claim (Section 3.4).

$$\frac{\begin{array}{c}\text{(The shapes of } \tau' \text{ and } \tau_2 \text{ are similar)}\\ \Theta \mid \mathcal{L} \mid \Gamma[x \hookleftarrow \tau_1][y \hookleftarrow (\tau_2 \wedge_y y =_{\tau_2} x)\,\mathbf{ref}^1] \vdash e : \tau \Rightarrow \Gamma'\end{array}}{\Theta \mid \mathcal{L} \mid \Gamma[x : \tau_1 + \tau_2][y : \tau'\,\mathbf{ref}^1] \vdash y := x\,;e : \tau \Rightarrow \Gamma'} \quad \text{(T-Assign)}$$

$$\frac{\begin{array}{c}(\tau_1\,\mathbf{ref}^{r_1} + \tau_2\,\mathbf{ref}^{r_2}) \approx (\tau_1'\,\mathbf{ref}^{r_1'} + \tau_2'\,\mathbf{ref}^{r_2'})\\ \Theta \mid \mathcal{L} \mid \Gamma[x \hookleftarrow \tau_1'\,\mathbf{ref}^{r_1'}][y \hookleftarrow \tau_2'\,\mathbf{ref}^{r_2'}] \vdash e : \tau \Rightarrow \Gamma'\end{array}}{\Theta \mid \mathcal{L} \mid \Gamma[x : \tau_1\,\mathbf{ref}^{r_1}][y : \tau_2\,\mathbf{ref}^{r_2}] \vdash \mathbf{alias}(x = y)\,;e : \tau \Rightarrow \Gamma'} \quad \text{(T-Alias)}$$

$$\frac{\begin{array}{c}(\tau_1\,\mathbf{ref}^{r_1} + \tau_2\,\mathbf{ref}^{r_2}) \approx (\tau_1'\,\mathbf{ref}^{r_1'} + \tau_2'\,\mathbf{ref}^{r_2'})\\ \Theta \mid \mathcal{L} \mid \Gamma[x \hookleftarrow \tau_1'\,\mathbf{ref}^{r_1}][y \hookleftarrow (\tau_2'\,\mathbf{ref}^{r_2'})\,\mathbf{ref}^{r}] \vdash e : \tau \Rightarrow \Gamma'\end{array}}{\Theta \mid \mathcal{L} \mid \Gamma[x : \tau_1\,\mathbf{ref}^{r_1}][y : (\tau_2\,\mathbf{ref}^{r_2})\,\mathbf{ref}^{r}] \vdash \mathbf{alias}(x = *y)\,;e : \tau \Rightarrow \Gamma'} \quad \text{(T-AliasPtr)}$$

$$\frac{\Gamma \leq \Gamma' \qquad \Theta \mid \mathcal{L} \mid \Gamma' \vdash e : \tau \Rightarrow \Gamma'' \qquad \Gamma'', \tau \leq \Gamma''', \tau'}{\Theta \mid \mathcal{L} \mid \Gamma \vdash e : \tau' \Rightarrow \Gamma'''} \quad \text{(T-Sub)}$$

$$\tau_1 \approx \tau_2 \text{ iff } \bullet \vdash \tau_1 \leq \tau_2 \text{ and } \bullet \vdash \tau_2 \leq \tau_1.$$

**Fig. 8.** Pointer manipulation and subtyping

$$\frac{\Gamma \models \varphi_1 \implies \varphi_2}{\Gamma \vdash \{\nu : \mathbf{int} \mid \varphi_1\} \leq \{\nu : \mathbf{int} \mid \varphi_2\}} \quad \text{(S-Int)} \qquad \frac{\forall\, x \in dom(\Gamma').\Gamma \vdash \Gamma(x) \leq \Gamma'(x)}{\Gamma \leq \Gamma'} \quad \text{(S-TyEnv)}$$

$$\frac{r_1 \geq r_2 \qquad \Gamma \vdash \tau_1 \leq \tau_2}{\Gamma \vdash \tau_1\,\mathbf{ref}^{r_1} \leq \tau_2\,\mathbf{ref}^{r_2}} \quad \text{(S-Ref)} \qquad \frac{\Gamma, x : \tau \leq \Gamma', x : \tau' \qquad x \notin dom(\Gamma)}{\Gamma, \tau \leq \Gamma, \tau'} \quad \text{(S-Res)}$$

**Fig. 9.** Subtyping rules.

*Destructive Updates, Aliasing, and Subtyping.* We now discuss the handling of assignment, aliasing annotations, and subtyping as described in Figure 8. Although apparently unrelated, all three concern updating the refinements of (potentially) aliased reference cells.

Like the binding forms discussed above, T-Assign splits the assigned value's type into two types via the type addition operator, and distributes these types between the right hand side of the assignment and the mutated reference contents. Refinement information in the fresh contents *may* be inconsistent with any previous refinement information; only the shapes must be the same. In a system with unrestricted aliasing, this typing rule would be unsound as it would admit writes that are inconsistent with refinements on aliases of the left hand side. However, the assignment rule requires that the updated reference has an ownership of 1. By the ownership type invariant, all aliases with the updated reference have 0 ownership, and by ownership well-formedness may only contain the $\top$ refinement.

*Example 3.* We can type the program as follows:

```
let x = mkref 5 in        // x:{ν:int | ν = 5} ref^1
let y = x in              // x : ⊤_1, y : {ν:int | ν = 5} ref^1
  y := 4; assert(*y = 4)  // x : ⊤_1, y : {ν:int | ν = 4} ref^1
```

In this and later examples, we include type annotations within comments. We stress that these annotations are for expository purposes only; our tool can infer these types automatically with no manual annotations.

As described thus far, the type system is quite strict: if ownership has been completely transferred from one reference to another, the refinement information found in the original reference is effectively useless. Additionally, once a mutable pointer has been split through an assignment or let expression, there is no way to recover mutability. The typing rule for must alias assertions, T-Alias and T-AliasPtr, overcomes this restriction by exploiting the must-aliasing information to “shuffle” or redistribute ownerships *and refinements* between two aliased pointers. The typing rule assigns two fresh types $\tau_1' \,\mathbf{ref}^{r_1'}$ and $\tau_2' \,\mathbf{ref}^{r_2'}$ to the two operand pointers. The choice of $\tau_1', r_1', \tau_2'$, and $r_2'$ is left open provided that the sum of the new types, $(\tau_1' \,\mathbf{ref}^{r_1'}) + (\tau_2' \,\mathbf{ref}^{r_2'})$ is equivalent (denoted $\approx$) to the sum of the original types. Formally, $\approx$ is defined as in Figure 8; it implies that any refinements in the two types must be logically equivalent and that ownerships must also be equal. This redistribution is sound precisely because the two references are assumed to alias; the total ownership for the single memory cell pointed to by both references cannot be increased by this shuffling. Further, any refinements that hold for the contents of one reference must necessarily hold for contents of the other and vice versa.

*Example 4 (Shuffling ownerships and refinements).* Let $\varphi_{=n}$ be $\nu = n$.

```
let x = mkref 5 in      // x:{ν:int | φ=5} ref^1
let y = x in            // x : ⊤1, y : {ν:int | φ=5} ref^1
  y := 4; alias(x = y)  // x : {ν:int | φ=4} ref^0.5, y : {ν:int | φ=4} ref^0.5
```

The final type assignment for $x$ and $y$ is justified by

$$\top_1 + \{\nu : \mathbf{int} \mid \varphi_{=4}\}\,\mathbf{ref}^1 = \{\nu : \mathbf{int} \mid \top \land \varphi_{=4}\}\,\mathbf{ref}^1 \approx$$
$$\{\nu : \mathbf{int} \mid \varphi_{=4} \land \varphi_{=4}\}\,\mathbf{ref}^1 = \{\nu : \mathbf{int} \mid \varphi_{=4}\}\,\mathbf{ref}^{0.5} + \{\nu : \mathbf{int} \mid \varphi_{=4}\}\,\mathbf{ref}^{0.5}\,.$$

The aliasing rules give fine-grained control over ownership information. This flexibility allows mutation through two or more aliased references within the same scope. Provided sufficient aliasing annotations, the type system may shuffle ownerships between one or more live references, enabling and disabling mutability as required. Although the reliance on these annotations appears to decrease the practicality of our type system, we expect these aliasing annotations can be inserted by a conservative must-aliasing analysis. Further, empirical experience from our prior work [56] indicates that only a small number of annotations are required for larger programs.

*Example 5 (Shuffling Mutability).* Let $\varphi_{=n}$ again be $\nu = n$. The following program uses two live, aliased references to mutate the same memory location:

```
let x = mkref 0 in
let y = x in              // x : {ν:int | φ=0} ref^1, y : ⊤1
  x := 1; alias(x = y);   // x : ⊤1, y : {ν:int | φ=1} ref^1
  y := 2; alias(x = y);   // x : {ν:int | φ=2} ref^0.5, y : {ν:int | φ=2} ref^0.5
  assert(*x = 2)
```

$$\frac{\begin{array}{c}\Theta(f) = \forall\lambda.\,\langle x_1 : \tau_1, \ldots, x_n : \tau_n\rangle \to \langle x_1 : \tau_1', \ldots, x_n : \tau_n' \mid \tau\rangle \\ \sigma_\alpha = [\ell : \mathcal{L}/\lambda] \qquad \sigma_x = [y_1/x_1]\cdots[y_n/x_n] \\ \Theta \mid \mathcal{L} \mid \Gamma[y_i \hookleftarrow \sigma_\alpha\,\sigma_x\,\tau_i'], x : \sigma_\alpha\,\sigma_x\,\tau \vdash e : \tau' \Rightarrow \Gamma' \qquad x \notin dom(\Gamma')\end{array}}{\Theta \mid \mathcal{L} \mid \Gamma[y_i : \sigma_\alpha\,\sigma_x\,\tau_i] \vdash \mathbf{let}\; x = f^\ell(y_1, \ldots, y_n)\;\mathbf{in}\; e : \tau' \Rightarrow \Gamma'} \quad \text{(T-Call)}$$

$$\frac{\begin{array}{c}\Theta(f) = \forall\lambda.\,\langle x_1 : \tau_1, \ldots, x_n : \tau_n\rangle \to \langle x_1 : \tau_1', \ldots, x_n : \tau_n' \mid \tau\rangle \\ \Theta \mid \lambda \mid x_1 : \tau_1, \ldots, x_n : \tau_n \vdash e : \tau \Rightarrow x_1 : \tau_1', \ldots, x_n : \tau_n'\end{array}}{\Theta \vdash f \mapsto (x_1, .., x_n)e} \quad \text{(T-FunDef)}$$

$$\frac{\begin{array}{c}\forall f \mapsto (x_1, .., x_n)e \in D.\Theta \vdash f \mapsto (x_1, .., x_n)e \\ dom(D) = dom(\Theta)\end{array}}{\Theta \vdash D} \quad \text{(T-Funs)} \qquad \frac{\begin{array}{c}\Theta \vdash D \qquad \vdash_{WF} \Theta \\ \Theta \mid \epsilon \mid \bullet \vdash e : \tau \Rightarrow \Gamma\end{array}}{\vdash \langle D, e\rangle} \quad \text{(T-Prog)}$$

**Fig. 10.** Program typing rules

After the first aliasing statement the type system shuffles the (exclusive) mutability between $x$ and $y$ to enable the write to $y$. After the second aliasing statement the ownership in $y$ is split with $x$; note that transferring all ownership from $y$ to $x$ would also yield a valid typing.

Finally, we describe the subtyping rule. The rules for subtyping types and environments are shown in Figure 9. For integer types, the rules require the refinement of a supertype is a logical consequence of the subtype's refinement conjoined with the lifting of $\Gamma$. The subtype rule for references is *covariant* in the type of reference contents. It is widely known that in a language with unrestricted aliasing and mutable references such a rule is unsound: after a write into the coerced pointer, reads from an alias may yield a value disallowed by the alias' type [43]. However, as in the assign case, ownership types prevent unsoundness; a write to the coerced pointer requires the pointer to have ownership 1, which guarantees any aliased pointers have the maximal type and provide no information about their contents beyond simple types.

#### 3.3 Interprocedural Fragment and Context-Sensitivity

We now turn to a discussion of the interprocedural fragment of our language, and how our type system propagates context information. The remaining typing rules for our language are shown in Figure 10. These rules concern the typing of function calls, function bodies, and entire programs.

We first explain the T-Call rule. The rule uses two substitution maps. $\sigma_x$ translates between the parameter names used in the function type and actual argument names at the call-site. $\sigma_\alpha$ instantiates all occurrences of $\lambda$ in the callee type with $\ell : \mathcal{L}$, where $\ell$ is the label of the call-site and $\mathcal{L}$ the typing context of the call. The types of the arguments $y_i$'s are required to match the parameter types (post substitution). The body of the let binding is then checked with the argument types updated to reflect the changes in the function call (again, post substitution). This update is well-defined because we require all function arguments be distinct as described in Section 2.1. Intuitively, the substitution $\sigma_\alpha$ represents incrementally refining the behavior of the callee function with partial context information. If $\mathcal{L}$ is itself a context variable $\lambda'$, this substitution effectively transforms any context prefix queries over $\lambda$ in the argument/return/output types into a queries over $\ell : \lambda'$. In other words, while the exact concrete execution context of the callee is unknown, the context must at least begin with $\ell$ which can potentially rule out certain behaviors.

Rule T-FunDef type checks a function definition $f \mapsto (x_1, .., x_n)e$ against the function type given in $\Theta$. As a convenience we assume that the parameter names in the function type match the formal parameters in the function definition. The rule checks that under an initial environment given by the argument types the function body produces a value of the return type and transforms the arguments according to the output types. As mentioned above, functions may be executed under many different contexts, so type checking the function body is performed under the context variable $\lambda$ that occurs in the function type.

Finally, the rule for typing programs (T-Prog) checks that all function definitions are well typed under a well-formed function type environment, and that the entry point $e$ is well typed in an empty type environment and the typing context $\epsilon$, i.e., the initial context.

*Example 6 (1-CFA).* Recall the program in Figure 3 in Section 1; assume the function calls are labeled as follows:

```
p := get^ℓ1(p) + 1;
// ...
q := get^ℓ2(q) + 1;
```

Taking $\tau_p$ to be the type shown in Example 2:

$$\{\nu : \mathbf{int} \mid (\ell_1 \preceq \lambda \implies \nu = 3) \land (\ell_2 \preceq \lambda \implies \nu = 5)\}$$

we can give `get` the type $\forall\lambda. \langle z : \tau_p\, \mathbf{ref}^1 \rangle \to \langle z : \tau_p\, \mathbf{ref}^1 \mid \tau_p \rangle$.

*Example 7 (2-CFA).* To see how context information propagates across multiple calls, consider the following change to the code considered in Example 6:

```
get_real(z) { *z }
get(z) { get_real^ℓ3(z) }
```

The type of `get` remains as in Example 6, and taking $\tau$ to be

$$\{\nu : \mathbf{int} \mid (\ell_3\, \ell_1 \preceq \lambda' \implies \nu = 3) \land (\ell_3\, \ell_2 \preceq \lambda' \implies \nu = 5)\}$$

the type of `get_real` is: $\forall\lambda'. \langle z : \tau\, \mathbf{ref}^1 \rangle \to \langle z : \tau\, \mathbf{ref}^1 \mid \tau \rangle$.

We focus on the typing of the call to `get_real` in `get`; it is typed in context $\lambda$ and a type environment where `p` is given type $\tau_p$ from Example 6.

Applying the substitution $[\ell_3 : \lambda/\lambda']$ to the argument type of `get_real` yields:

$$\{\nu : \mathbf{int} \mid (\ell_3\,\ell_1 \preceq \ell_3 : \lambda \implies \nu = 3) \wedge (\ell_3\,\ell_2 \preceq \ell_3 : \lambda \implies \nu = 5)\}\,\mathbf{ref}^1 \approx$$
$$\{\nu : \mathbf{int} \mid (\ell_1 \preceq \lambda \implies \nu = 3) \wedge (\ell_2 \preceq \lambda \implies \nu = 5)\}\,\mathbf{ref}^1$$

which is exactly the type of `p`. A similar derivation applies to the return type of `get_real` and thus `get`.

#### 3.4 Soundness

We have proven that any program that type checks according to the rules above will never experience an assertion failure. We formalize this claim with the following soundness theorem.

**Theorem 1 (Soundness).** *If* $\vdash \langle D, e\rangle$, *then* $\langle \emptyset, \emptyset, \cdot, e\rangle \not\longrightarrow^*_D$ **AssertFail**.

*Further, any well-typed program either diverges, halts in the configuration* **AliasFail**, *or halts in a configuration* $\langle H, R, \cdot, x\rangle$ *for some* $H, R$ *and* $x$, *i.e., evaluation does not get stuck.*

*Proof (Sketch).* By standard progress and preservation lemmas; the full proof has been omitted for space reasons and can be found in the full version [60].

### 4 Inference and Extensions

We now briefly describe the inference algorithm implemented in our tool ConSORT. We sketch some implemented extensions needed to type more interesting programs and close with a discussion of current limitations of our prototype.

#### 4.1 Inference

Our tool first runs a standard, simple type inference algorithm to generate type templates for every function parameter type, return type, and for every live variable at each program point. For a variable $x$ of simple type $\tau_S ::= \mathbf{int} \mid \tau_S\,\mathbf{ref}$ at program point $p$, ConSORT generates a type template $[\![\tau_S]\!]_{x,0,p}$ as follows:

$$[\![\mathbf{int}]\!]_{x,n,p} = \{\nu : \mathbf{int} \mid \varphi_{x,n,p}(\nu; \mathbf{FV}_p)\} \quad [\![\tau_S\,\mathbf{ref}]\!]_{x,n,p} = [\![\tau_S]\!]_{x,n+1,p}\,\mathbf{ref}^{r_{x,n,p}}$$

$\varphi_{x,n,p}(\nu; \mathbf{FV}_p)$ denotes a fresh relation symbol applied to $\nu$ and the free variables of simple type **int** at program point $p$ (denoted $\mathbf{FV}_p$). $r_{x,n,p}$ is a fresh ownership variable. For each function $f$, there are two synthetic program points, $f^b$ and $f^e$ for the beginning and end of the function respectively. At both points, ConSORT generates type template for each argument, where $\mathbf{FV}_{f^b}$ and $\mathbf{FV}_{f^e}$ are the names of integer typed parameters. At $f^e$, ConSORT also generates a type template for the return value. We write $\Gamma^p$ to indicate the type environment at point $p$, where every variable is mapped to its corresponding type template. $[\![\Gamma^p]\!]$ is thus equivalent to $\bigwedge_{x \in \mathbf{FV}_p} \varphi_{x,0,p}(x; \mathbf{FV}_p)$.

When generating these type templates, our implementation also generates ownership well-formedness constraints. Specifically, for a type template of the form $\{\nu:\mathbf{int} \mid \varphi_{x,n+1,p}(\nu;\mathbf{FV}_p)\}\,\mathbf{ref}^{r_{x,n,p}}$ ConSORT emits the constraint: $r_{x,n,p} = 0 \implies \varphi_{x,n+1,p}(\nu;\mathbf{FV}_p)$ and for a type template $(\tau\,\mathbf{ref}^{r_{x,n+1,p}})\,\mathbf{ref}^{r_{x,n,p}}$ ConSORT emits the constraint $r_{x,n,p} = 0 \implies r_{x,n+1,p} = 0$.

ConSORT then walks the program, generating constraints between relation symbols and ownership variables according to the typing rules. These constraints take three forms, ownership constraints, subtyping constraints, and assertion constraints. Ownership constraints are simple linear (in)equalities over ownership variables and constants, according to conditions imposed by the typing rules. For example, if variable $x$ has the type template $\tau\,\mathbf{ref}^{r_{x,0,p}}$ for the expression $x := y\,;e$ at point $p$, ConSORT generates the constraint $r_{x,0,p} = 1$.

ConSORT emits subtyping constraints between the relation symbols at related program points according to the rules of the type system. For example, for the term $\mathbf{let}\, x = y \,\mathbf{in}\, e$ at program point $p$ (where $e$ is at program point $p'$, and $x$ has simple type $\mathbf{int}\,\mathbf{ref}$) ConSORT generates the following subtyping constraint:

$$[\![\Gamma^p]\!] \wedge \varphi_{y,1,p}(\nu;\mathbf{FV}_p) \implies \varphi_{y,1,p'}(\nu;\mathbf{FV}_{p'}) \wedge \varphi_{x,1,p'}(\nu;\mathbf{FV}_{p'})$$

in addition to the ownership constraint $r_{y,0,p} = r_{y,0,p'} + r_{x,0,p'}$.

Finally, for each $\mathbf{assert}(\varphi)$ in the program, ConSORT emits an assertion constraint of the form: $[\![\Gamma^p]\!] \implies \varphi$ which requires the refinements on integer typed variables in scope are sufficient to prove $\varphi$.

*Encoding Context Sensitivity.* To make inference tractable, we require the user to fix *a priori* the maximum length of prefix queries to a constant $k$ (this choice is easily controlled with a command line parameter to our tool). We supplement the arguments in *every* predicate application with a set of integer context variables $c_1, \ldots, c_k$; these variables do not overlap with any program variables.

ConSORT uses these variables to infer context sensitive refinements as follows. Consider a function call $\mathbf{let}\, x = f^{\ell}(y_1, \ldots, y_n)\,\mathbf{in}\, e$ at point $p$ where $e$ is at point $p'$. ConSORT generates the following constraint for a refinement $\varphi_{y_i,n,p}(\nu, c_1, \ldots, c_k;\mathbf{FV}_p)$ which occurs in the type template of $y_i$:

$$\begin{aligned}
&\varphi_{y_i,n,p}(\nu, c_0, \ldots, c_k;\mathbf{FV}_p) \implies \sigma_x\, \varphi_{x_i,n,f^b}(\nu, \ell, c_0, \ldots, c_{k-1};\mathbf{FV}_{f^b})\\
&\sigma_x\, \varphi_{x_i,n,f^e}(\nu, \ell, c_0, \ldots, c_{k-1};\mathbf{FV}_{f^e}) \implies \varphi_{y_i,n,p'}(\nu, c_0, \ldots, c_k;\mathbf{FV}_{p'})\\
&\sigma_x = [y_1/x_1]\cdots[y_n/x_n]
\end{aligned}$$

Effectively, we have encoded $\ell_1 \ldots \ell_k \preceq \lambda$ as $\wedge_{0<i\leq k} c_i = \ell_i$. In the above, the shift from $c_0, \ldots, c_k$ to $\ell, c_0, \ldots, c_{k-1}$ plays the role of $\sigma_\alpha$ in the T-Call rule. The above constraint serves to determine the value of $c_0$ within the body of the function $f$. If $f$ calls another function $g$, the above rule propagates this value of $c_0$ to $c_1$ within $g$ and so on. The solver may then instantiate relation symbols with predicates that are conditional over the values of $c_i$.

*Solving Constraints.* The results of the above process are two systems of constraints; real arithmetic constraints over ownership variables and constrained Horn clauses (CHC) over the refinement relations. Under certain assumptions about the simple types in a program, the size of the ownership and subtyping constraints will be polynomial to the size of the program. These systems are not independent; the relation constraints may mention the value of ownership variables due to the well-formedness constraints described above. The ownership constraints are first solved with Z3 [16]. These constraints are non-linear but Z3 appears particularly well-engineered to quickly find solutions for the instances generated by ConSORT. We constrain Z3 to maximize the number of non-zero ownership variables to ensure as few refinements as possible are constrained to be $\top$ by ownership well-formedness.

The values of ownership variables inferred by Z3 are then substituted into the constrained Horn clauses, and the resulting system is checked for satisfiability with an off-the-shelf CHC solver. Our implementation generates constraints in the industry standard SMT-Lib2 format [8]; any solver that accepts this format can be used as a backend for ConSORT. Our implementation currently supports Spacer [37] (part of the Z3 solver [16]), HoICE [13], and Eldarica [48] (adding a new backend requires only a handful of lines of glue code). We found that different solvers are better tuned to different problems; we also implemented *parallel mode* which runs all supported solvers in parallel, using the first available result.

#### 4.2 Extensions

*Primitive Operations.* As defined in Section 2, our language can compare integers to zero and load and store them from memory, but can perform no meaningful computation over these numbers. To promote the flexibility of our type system and simplify our soundness statement, we do not fix a set of primitive operations and their static semantics. Instead, we assume any set of primitive operations used in a program are given sound function types in $\Theta$. For example, under the assumption that $+$ has its usual semantics and the underlying logic supports $+$, we can give $+$ the type $\forall\lambda.\,\langle x : \top_0, y : \top_0\rangle \to \langle x : \top_0, y : \top_0 \mid \{\nu : \mathbf{int} \mid \nu = x + y\}\rangle$. Interactions with a nondeterministic environment or unknown program inputs can then be modeled with a primitive that returns integers refined with $\top$.

*Dependent Tuples.* Our implementation supports types of the form: $(x_1 : \tau_1, \ldots, x_n : \tau_n)$, where $x_i$ can appear within $\tau_j$ $(j \neq i)$ if $\tau_i$ is an integer type. For example, $(x : \{\nu : \mathbf{int} \mid \top\}, y : \{\nu : \mathbf{int} \mid \nu > x\})$ is the type of tuples whose second element is strictly greater than the first. We also extend the language with tuple constructors as a new value form, and let bindings with tuple patterns as the LHS.

The extension to type checking is relatively straightforward; the only significant extensions are to the subtyping rules. Specifically, the subtyping check for a tuple element $x_i : \tau_i$ is performed in a type environment elaborated with the types and names of other tuple elements. The extension to type inference is also straightforward; the arguments for a predicate symbol include any enclosing dependent tuple names and the environment in subtyping constraints is likewise extended.

*Recursive Types.* Our language also supports some unbounded heap structures via recursive reference types. To keep inference tractable, we forbid nested recursive types, multiple occurrences of the recursive type variable, and additionally fix the shape of refinements that occur within a recursive type. For recursive refinements that fit the above restriction, our approach for refinements is broadly similar to that in [35], and we use the ownership scheme of [56] for handling ownership. We first use simple type inference to infer the shape of the recursive types, and automatically insert fold/unfold annotations into the source program. As in [35], the refinements within an unfolding of a recursive type may refer to dependent tuple names bound by the enclosing type. These recursive types can express, e.g., the invariants of a mutable, sorted list. As in [56], recursive types are unfolded once before assigning ownership variables; further unfoldings copy existing ownership variables.

As in Java or C++, our language does not support sum types, and any instantiation of a recursive type must use a null pointer. Our implementation supports an **ifnull** construct in addition to a distinguished **null** constant. Our implementation allows any refinement to hold for the null constant, including $\bot$. Currently, our implementation does *not* detect null pointer dereferences, and all soundness guarantees are made modulo freedom of null dereferences. As $[\![\Gamma]\!]$ omits refinements under reference types, null pointer refinements do not affect the verification of programs without null pointer dereferences.

*Arrays.* Our implementation supports arrays of integers. Each array is given an ownership describing the ownership of memory allocated for the entire array. The array type contains two refinements: the first refines the length of the array itself, and the second refines the entire array contents. The content refinement may refer to a symbolic index variable for precise, per-index refinements. At reads and writes to the array, ConSORT instantiates the refinement's symbolic index variable with the concrete index used at the read/write.

As in [56], our restriction to arrays of integers stems from the difficulty of ownership inference. Soundly handling pointer arrays requires index-wise tracking of ownerships which significantly complicates automated inference. We leave supporting arrays of pointers to future work.

#### 4.3 Limitations

Our current approach is not complete; there are safe programs that will be rejected by our type system. As mentioned in Section 3.1, our well-formedness condition forbids refinements that refer to memory locations. As a result, ConSORT cannot in general express, e.g., that the contents of two references are equal. Further, due to our reliance on automated theorem provers we are restricted to logics with sound but potentially incomplete decision procedures. ConSORT also does not support conditional or context-sensitive ownerships, and therefore cannot precisely handle conditional mutation or aliasing.

### 5 Experiments

We now present the results of preliminary experiments performed with the implementation described in Section 4. The goal of these experiments was to answer the

**Table 1.** Description of benchmark suite adapted from JayHorn. **Java** are programs that test Java-specific features. **Inc** are tests that cannot be handled by ConSORT, e.g., null checking, etc. **Bug** includes a "safe" program we discovered was actually incorrect.

| Set | Orig. | Adapted | Java | Inc | Bug |
|---|---|---|---|---|---|
| Safe | 41 | 32 | 6 | 2 | 1 |
| Unsafe | 41 | 26 | 13 | 2 | 0 |

following questions: i) is the type system (and extensions of Section 4) expressive enough to type and verify non-trivial programs? and ii) is type inference feasible?

To answer these questions, we evaluated our prototype implementation on two sets of benchmarks.[4] The first set is adapted from JayHorn [32, 33], a verification tool for Java. This test suite contains a combination of 82 safe and unsafe programs written in Java. We chose this benchmark suite as, like ConSORT, JayHorn is concerned with the automated verification of programs in a language with mutable, aliased memory cells. Further, although some of their benchmark programs tested Java specific features, most could be adapted into our low-level language. The tests we could adapt provide a comparison with existing state-of-the-art verification techniques. A detailed breakdown of the adapted benchmark suite can be found in Table 1.

*Remark 2.* The original JayHorn paper includes two additional benchmark sets, Mine Pump and CBMC. Both our tool and recent JayHorn versions time out on the Mine Pump benchmark. Further, the CBMC tests were either subsumed by our own test programs, tested Java specific features, or tested program synthesis functionality. We therefore omitted both of these benchmarks from our evaluation.

The second benchmark set consists of data structure implementations and microbenchmarks written directly in our low-level imperative language. We developed this suite to test the expressive power of our type system and inference. The programs included in this suite are:

- **Array-List** Implementation of an unbounded list backed by an array.
- **Sorted-List** Implementation of a mutable, sorted list maintained with an in-place insertion sort algorithm.
- **Shuffle** Multiple live references are used to mutate the same location in program memory as in Example 5.
- **Mut-List** Implementation of general linked lists with a clear operation.
- **Array-Inv** A program which allocates a length $n$ array and writes the value $i$ at every index $i$.
- **Intro2** The motivating program shown in Figure 2 in Section 1.

[4] Our experiments and the ConSORT source code are available at https://www.fos.kuis.kyoto-u.ac.jp/projects/consort/.

**Table 2.** Comparison of ConSORT to JayHorn on the benchmark set of [32] (top) and our custom benchmark suite (bottom). *T/O* indicates a time out.

| | | **ConSORT** | | **JayHorn** | | |
|---|---|---|---|---|---|---|
| **Set** | **N. Tests** | *Correct* | *T/O* | *Correct* | *T/O* | *Imp.* |
| **Safe** | 32 | 29 | 3 | 24 | 5 | 3 |
| **Unsafe** | 26 | 26 | 0 | 19 | 0 | 7 |

| **Name** | **Safe?** | **Time(s)** | **Ann** | **JH** | **Name** | **Safe?** | **Time(s)** | **Ann** | **JH** |
|---|---|---|---|---|---|---|---|---|---|
| **Array-Inv** | ✓ | 10.07 | 0 | T/O | **Array-Inv-BUG** | X | 5.29 | 0 | T/O |
| **Array-List** | ✓ | 16.76 | 0 | T/O | **Array-List-BUG** | X | 1.13 | 0 | T/O |
| **Intro2** | ✓ | 0.08 | 0 | T/O | **Intro2-BUG** | X | 0.02 | 0 | T/O |
| **Mut-List** | ✓ | 1.45 | 3 | T/O | **Mut-List-BUG** | X | 0.41 | 3 | T/O |
| **Shuffle** | ✓ | 0.13 | 3 | ✓ | **Shuffle-BUG** | X | 0.07 | 3 | X |
| **Sorted-List** | ✓ | 1.90 | 3 | T/O | **Sorted-List-BUG** | X | 1.10 | 3 | T/O |

We introduced unsafe mutations to these programs to check our tool for unsoundness and translated these programs into Java for further comparison with JayHorn.

Our benchmarks and JayHorn's require a small number of trivially identified alias annotations. The adapted JayHorn benchmarks contain a total of 6 annotations; the most for any individual test was 3. The number of annotations required for our benchmark suite are shown in column **Ann.** of Table 2.

We first ran ConSORT on each program in our benchmark suite and ran version 0.7 of JayHorn on the corresponding Java version. We recorded the final verification result for both our tool and JayHorn. We also collected the end-to-end runtime of ConSORT for each test; we do not give a performance comparison with JayHorn given the many differences in target languages. For the JayHorn suite, we first ran our tool on the adapted version of each test program and ran JayHorn on the original Java version. We also did not collect runtime information for this set of experiments because our goal is a comparison of tool precision, not performance. All tests were run on a machine with 16 GB RAM and 4 Intel i5 CPUs at 2GHz and with a timeout of 60 seconds (the same timeout was used in [32]). We used ConSORT's parallel backend (Section 4) with Z3 version 4.8.4, HoICE version 1.8.1, and Eldarica version 2.0.1 and JayHorn's Eldarica backend.

#### 5.1 Results

The results of our experiments are shown in Table 2. On the JayHorn benchmark suite ConSORT performs competitively with JayHorn, correctly identifying 29 of the 32 safe programs as such. For all 3 tests on which ConSORT timed out after 60 seconds, JayHorn also timed out (column *T/O*). For the unsafe programs, ConSORT correctly identified all programs as unsafe within 60 seconds; JayHorn answered Unknown for 7 tests (column *Imp.*).

On our own benchmark set, ConSORT correctly verifies all safe versions of the programs within 60 seconds. For the unsafe variants, ConSORT was able to quickly and definitively determine these programs unsafe. JayHorn times out on all tests except for **Shuffle** and **ShuffleBUG** (column **JH**). We investigated the cause of time outs and discovered that after verification failed with an unbounded heap model, JayHorn attempts verification on increasingly larger bounded heaps. In every case, JayHorn exceeded the 60 second timeout before reaching a preconfigured limit on the heap bound. This result suggests JayHorn struggles in the presence of per-object invariants and unbounded allocations; the only two tests JayHorn successfully analyzed contain just a single object allocation.

We do not believe this struggle is indicative of a shortcoming in JayHorn's implementation, but stems from the fundamental limitations of JayHorn's memory representation. Like many verification tools (see Section 6), JayHorn uses a single, unchanging invariant to for every object allocated at the same syntactic location; effectively, all objects allocated at the same location are assumed to alias with one another. This representation cannot, in general, handle programs with different invariants for distinct objects that evolve over time. We hypothesize other tools that adopt a similar approach will exhibit the same difficulty.

### 6 Related Work

The difficulty in handling programs with mutable references and aliasing has been well-studied. Like JayHorn, many approaches model the heap explicitly at verification time, approximating concrete heap locations with allocation site labels [14, 20, 32, 33, 46]; each *abstract location* is also associated with a refinement. As abstract locations summarize many concrete locations, this approach does not in general admit strong updates and flow-sensitivity; in particular, the refinement associated with an abstract location is fixed for the lifetime of the program. The techniques cited above include various workarounds for this limitation. For example, [14, 46] temporarily allows breaking these invariants through a distinguished program name as long as the abstract location is not accessed through another name. The programmer must therefore eventually bring the invariant back in sync with the summary location. As a result, these systems ultimately cannot precisely handle programs that require evolving invariants on mutable memory.

A similar approach was taken in CQual [23] by Aiken et al. [2]. They used an explicit *restrict* binding for pointers. Strong updates are permitted through pointers bound with *restrict*, but the program is forbidden from using any pointers which share an allocation site while the restrict binding is live.

A related technique used in the field of object-oriented verification is to declare object invariants at the class level and allow these invariants on object fields to be broken during a limited period of time [7, 22]. In particular, the work on Spec# [7] uses an ownership system which tracks whether object $a$ owns object $b$; like ConSORT's ownership system, these ownerships contain the effects of mutation. However, Spec#'s ownership is quite strict and does not admit references to $b$ outside of the owning object $a$.

Viper [30, 42] (and its related projects [31, 39]) uses access annotations (expressed as permission predicates) to explicitly transfer access/mutation permissions for references between static program names. Like ConSORT, permissions may be fractionally transferred, allowing temporary shared, immutable access to a mutable memory cell. However, while ConSORT automatically infers many ownership transfers, Viper requires extensive annotations for each transfer.

F*, a dependently typed dialect of ML, includes an update/select theory of heaps and requires explicit annotations summarizing the heap effects of a method [44, 57, 58]. This approach enables modular reasoning and precise specification of pre- and post-conditions with respect to the heap, but precludes full automation.

The work on rely–guarantee reference types by Gordon et al. [26, 27] uses refinement types in a language mutable references and aliasing. Their approach extends reference types with rely/guarantee predicates; the rely predicate describes possible mutations via aliases, and the guarantee predicate describes the admissible mutations through the current reference. If two references may alias, then the guarantee predicate of one reference implies the rely predicate of the other and vice versa. This invariant is maintained with a splitting operation that is similar to our + operator. Further, their type system allows strong updates to reference refinements provided the new refinements are preserved by the rely predicate. Thus, rely–guarantee refinement support multiple mutable, aliased references with non-trivial refinement information. Unfortunately this expressiveness comes at the cost of automated inference and verification; an embedding of this system into Liquid Haskell [63] described in [27] was forced to sacrifice strong updates.

Work by Degen et al. [17] introduced linear *state annotations* to Java. To effect strong updates in the presence of aliasing, like ConSORT, their system requires annotated memory locations are mutated only through a distinguished reference. Further, all aliases of this mutable reference give no information about the state of the object much like our 0 ownership pointers. However, their system cannot handle multiple, immutable aliases with non-trivial annotation information; *only* the mutable reference may have non-trivial annotation information.

The fractional ownerships in ConSORT and their counterparts in [55, 56] have a clear relation to linear type systems. Many authors have explored the use of linear type systems to reason in contexts with aliased mutable references [18, 19, 52], and in particular with the goal of supporting strong updates [1]. A closely related approach is RustHorn by Matsushita et al. [40]. Much like ConSORT, RustHorn uses CHC and linear aliasing information for the sound and—unlike ConSORT—complete verification of programs with aliasing and mutability. However, their approach depends on Rust's strict *borrowing discipline*, and cannot handle programs where multiple aliased references are used in the same lexical region. In contrast, ConSORT supports fine-grained, per-statement changes in mutability and even further control with **alias** annotations, which allows it to verify larger classes of programs.

The ownerships of ConSORT also have a connection to separation logic [45]; the separating conjunction isolates write effects to local subheaps, while ConSORT's ownership system isolates effects to local updates of pointer types. Other researchers have used separation logic to precisely support strong updates of abstract state. For example, in work by Kloos et al. [36] resources are associated with static, abstract names; each resource (represented by its static name) may be owned (and thus, mutated) by exactly one thread. Unlike ConSORT, their ownership system forbids even temporary immutable, shared ownership, or transferring ownerships at arbitrary program points. An approach proposed by Bakst and Jhala [4] uses a similar technique, combining separation logic with refinement types. Their approach gives allocated memory cells abstract names, and associates these names with refinements in an abstract heap. Like the approach of Kloos et al. and ConSORT's ownership 1 pointers, they ensure these abstract locations are distinct in all concrete heaps, enabling sound, strong updates.

The idea of using a rational number to express permissions to access a reference dates back to the type system of *fractional permissions* by Boyland [12]. His work used fractional permissions to verify race freedom of a concurrent program without a may-alias analysis. Later, Terauchi [59] proposed a type-inference algorithm that reduces typing constraints to a set of linear inequalities over rational numbers. Boyland's idea also inspired a variant of separation logic for a concurrent programming language [11] to express sharing of read permissions among several threads. Our previous work [55, 56], inspired by that in [11, 59], proposed methods for type-based verification of resource-leak freedom, in which a rational number expresses an *obligation* to deallocate certain resource, not just a permission.

The issue of context-sensitivity (sometimes called *polyvariance*) is well-studied in the field of abstract interpretation (e.g., [28, 34, 41, 50, 51], see [25] for a recent survey). Polyvariance has also been used in type systems to assign different behaviors to the same function depending on its call site [3, 6, 64]. In the area of refinement type systems, Zhu and Jagannathan developed a context-sensitive dependent type system for a functional language [67] that indexed function types by unique labels attached to call-sites. Our context-sensitivity approach was inspired by this work. In fact, we could have formalized context-polymorphism within the framework of full dependent types, but chose the current presentation for simplicity.

### 7 Conclusion

We presented ConSORT, a novel type system for safety verification of imperative programs with mutability and aliasing. ConSORT is built upon the novel combination of fractional ownership types and refinement types. Ownership types flow-sensitively and precisely track the existence of mutable aliases. ConSORT admits sound strong updates by discarding refinement information on mutably-aliased references as indicated by ownership types. Our type system is amenable to automatic type inference; we have implemented a prototype of this inference tool and found it can verify several non-trivial programs and outperforms a state-of-the-art program verifier. As an area of future work, we plan to investigate using fractional ownership types to soundly allow refinements that mention memory locations.

*Acknowledgments* The authors would like to the reviewers for their thoughtful feedback and suggestions, and Yosuke Fukuda and Alex Potanin for their feedback on early drafts. This work was supported in part by JSPS KAKENHI, grant numbers JP15H05706 and JP19H04084, and in part by the JST ERATO MMSD Project.
